# The Nature of Lexical-Semantic Access in Bilingual Aphasia

**DOI:** 10.1155/2014/389565

**Published:** 2014-03-30

**Authors:** Swathi Kiran, Isabel Balachandran, Jason Lucas

**Affiliations:** Department of Speech Language and Hearing Sciences, Boston University Sargent College, 635 Commonwealth Avenue, Boston, MA 02215, USA

## Abstract

*Background.* Despite a growing clinical need, there are no clear guidelines on assessment of lexical access in the two languages in individuals with bilingual aphasia. *Objective.* In this study, we examined the influence of language proficiency on three tasks requiring lexical access in English and Spanish bilingual normal controls and in bilingual individuals with aphasia. *Methods.* 12 neurologically healthy Spanish-English bilinguals and 10 Spanish-English bilinguals with aphasia participated in the study. All participants completed three lexical retrieval tasks: two picture-naming tasks (BNT, BPNT) and a category generation (CG) task. *Results.* This study found that across all tasks, the greatest predictors for performance were the effect of group and language ability rating (LAR). Bilingual controls had a greater score or produced more correct responses than participants with bilingual aphasia across all tasks. The results of our study also indicate that normal controls and bilinguals with aphasia make similar types of errors in both English and Spanish and develop similar clustering strategies despite significant performance differences between the groups. *Conclusions.* Differences between bilingual patients and controls demonstrate a fundamental lexical retrieval deficit in bilingual individuals with aphasia, but one that is further influenced by language proficiency in the two languages.

## 1. Introduction

Naming deficits are a commonly acquired disorder, manifesting in all types of aphasia [1, 2]; however, we are still unclear about the nature and mechanisms underlying lexical processing deficits in monolingual and bilingual individuals with aphasia. Theories of normal bilingual language processing indicate variable degrees of overlap between the two languages. For instance, the* revised hierarchical model* (RHM; [3–5]) allows for language proficiency differences by proposing connections between both L1 and L2 and the semantic system; these connections differ in their strengths as a function of fluency in L1 relative to L2. In bilingual individuals with a dominant language, the lexicon of L1 is generally assumed to be larger than that of L2 because more words are known in the dominant language. Also, lexical associations from L2 to L1 are assumed to be stronger than those from L1 to L2. Conversely, the links between the semantic system and L1 are assumed to be stronger than from the semantic system to L2. With regards to activation of phonological representations from the semantic system, the prevailing theory suggests that activation flows from the semantic system to the phonological system of both languages simultaneously, indicating that lexical access is target language-nonspecific [6, 7]. Thus, targets in both languages are potentially active subsequent to semantic activation, but through a process of competitive selection, the target in the accurate language is ultimately produced. An alternate, but not necessarily contradictory hypothesis, is the fact that in order for bilinguals to access the target language, the nontarget language must be inhibited [8–10]. In other words, a speaker activates target language lemmas while simultaneously inhibiting the lemmas of the nontarget language.

There are several methods to examine lexical access in bilingual individuals. The most common approach has been confrontation picture naming. In general, performance on picture naming tasks is constrained by the images presented and influenced by word frequency and imageability. One such picture naming task that has been used extensively as a measure of lexical access in monolinguals and bilinguals is the Boston Naming Test (BNT, [11]). For instance, Kohnert et al. [12] showed that normal young bilinguals performed better in English than Spanish on the BNT and that naming accuracy significantly correlated with self-ratings of language skills. Similarly, Roberts et al. [13] examined naming on the BNT in French/English and Spanish/English bilinguals and found that both bilingual groups scored significantly below the monolingual English group on the BNT.

Another approach to examining lexical access includes category generation verbal fluency tasks [14–17]. Verbal fluency has been found to be dependent on a multitude of factors, including two qualitative features, clustering and switching ability. These strategic processes are mediated by executive functioning and verbal memory storage and have therefore been a successful predictor of lexical access ability [18, 19]. Performance on the task is highly contingent on the success of the generation of semantically related words in a subcategory, or clustering, which utilizes an individual's language stores. There is also an equally essential component of switching between subordinate categories in the verbal fluency task, which relies on an efficient cognitive flexibility [20–22]. Therefore, simply examining the number of correct words is not sufficient to understand the performance on the task [16].

The nature of semantic organization in the two languages of a bilingual individual affects influences their performance on verbal fluency tasks. For instance, Roberts and Le Dorze [23] examined category generation in French-English participants and found that there was no language effect on the number of correct responses across languages. However, for* animals*, French-English bilinguals recalled more subcategories (*birds, insects, etc.*) in French than English. The authors suggested that some semantic fields may have similar type of semantic organization across languages, whereas others may differ between languages even in balanced bilinguals. The authors suggested that childhood experiences and the cultural environment play an important role in determining the nature of semantic system.

In another set of studies, Rosselli et al. [24] first compared Spanish-English bilinguals with English monolinguals and Spanish monolinguals on word fluency task using either phoneme letter cues or semantic categories. Results showed a lower performance in the bilingual participants compared to their monolingual counterparts on the semantic category cued task but not on the phoneme letter cue task. They indicated that the shared elements of concrete nouns across languages may further the interference between the two languages. There may also be a greater conflict between the languages while the individual is searching through their verbal stores for semantically related words [24]. Interestingly, age of acquisition of L2 did interact with language, bilinguals who learned English earlier in life as L2 performed significantly higher than later learners on English versions of the tests. In a follow-up study, Rosselli et al. [25] examined the use of grammatical words versus content words for phonemic word generation and analyzed the relationship between productivity and semantic association for the responses in category generation. Results for generation of words within phonemic categories were similar to the previous study [24] in which bilinguals produced almost an identical number of words as both English and Spanish monolinguals. There are other studies that have examined verbal fluency as a measure of lexical-semantic access in bilingual individuals in other language combinations (e.g., Zulu/English, [26]; Finnish/English, [27]) and found differences in the degree of performance across the two languages of the bilingual. To summarize, most studies examining category fluency in bilingual individuals have demonstrated that participants tend to produce more items in one language relative to another and to task set (e.g., semantic or phonological cues), but no study has systematically examined the nature of category fluency in bilingual individuals across a set of semantic categories by taking into account language proficiency.

Both lexical access tasks described above, picture naming and verbal fluency, test lexical access but in slightly different ways. In both tasks, the measure of lexical access theoretically involves parallel activation of both languages with highly interactive phonological and semantic representations that spread through the levels of language representation [6]. However, sufficient crucial differences in the theoretical basis between the tasks exist to investigate different properties of lexical access. Performance on picture naming tasks is constrained by the images presented, making nonlinguistic strategies like clustering and switching ineffective. Performance on the picture naming tasks is driven mainly by word frequency and imageability. Also, categories in the category generation task have a certain degree of flexibility with regard to items that belong to a given category which is not present in a picture naming task. On the verbal fluency task, however, nonlinguistic and semantically unrelated phonological strategies are effective means of performing the task. Grouping clusters is dependent on the way semantically related words are organized in the brain. Clustering and switching abilities on the verbal fluency task are dependent on individual language exposure. The relative freedom of the category generation task (to semantically organize the categories) also aids in the performance of the task by facilitating the individual language abilities of the participants.

In contrast to studies on lexical access in nonbrain damaged bilingual individuals, examination of lexical access in bilingual aphasia is relatively sparse and most studies are case studies of individuals with interesting but atypical language impairment profiles [11, 28–33]. In one group study, Tschirren et al. [34] examined the interaction of late age of acquisition (AoA) on L2 syntactic deficits in bilingual aphasia. A total of 12 late bilingual patients with aphasia (six with anterior lesions and six with posterior lesions) were examined. The authors found that, as a group, the L1 and L2 aphasia severity scores did not differ; however, four patients with lesions in the prerolandic area did exhibit lower scores in L2 syntactic processing compared to L1 syntactic processing.

A few studies have specifically examined lexical access in bilingual aphasia. For instance, Roberts and Deslauriers [35] examined the relationship between the mental representation of the two languages and how effectively individuals switched between languages. During naming performance on cognate nouns, the study found that bilingual individuals with aphasia produced cognate nouns with higher accuracy than noncognates in both languages. In another study, Muñoz and Marquardt [36] compared language history and language proficiency self-ratings with poststroke picture naming and identification ability in four Spanish-English patients with bilingual aphasia with 20 neurologically healthy Spanish-English adults who were gender, ethnicity, and age matched and completed the same experiment diagnostics. The bilingual nonbrain damaged individuals showed that more frequent use of the English language is consistent with between-language differences in proficiency and literacy. The four patients fell into three patterns. For two patients differences in naming and identification scores in Spanish and English were correlated with varying degrees of skill between two languages instead of a differential impairment. For a third patient, it was predicted that his performance in English would outperform Spanish based on the language history; however, this trend was not observed and the authors identified a differential impairment. Finally, the fourth patient presented with a language profile that predicted similar impairments across languages; however, the English picture naming task was less impaired than the Spanish whereas the opposite trend in results was observed for the picture identification task. For this patient, the authors speculate that higher English picture naming scores may be attributed to strategies learned in years of English therapy that did not transfer to Spanish. Overall, the experiment results strongly suggest that an in-depth premorbid language history is a vital piece to the evaluation and identification of deficits and language pattern impairments in bilingual aphasia.

These studies highlight the fact that lexical retrieval is influenced by proficiency and the nature of brain damage, but these results are not necessarily generalizable to the larger population of bilingual aphasia. A systematic examination of a larger group of patients on different language tasks while accounting for language proficiency will help better understand the nature of lexical access in individuals with bilingual aphasia and guide better diagnosis and treatment of lexical impairment in these individuals.

The present study examines lexical access in English and Spanish with respect to both premorbid proficiency and the effect of stroke on language ability in ten patients with bilingual aphasia and their nonbrain damaged controls. We compared picture naming on the BNT with a separate normed naming task to examine any differences (or similarities) between these two tasks. While the BNT is used often in the assessment of lexical impairment in individuals with bilingual aphasia, it has clear limitations as a valid measure of lexical access due to the relatively low frequency of certain items in the task [27]. Therefore, in the present study, we directly compared performance on the BNT with another naming task that developed to examine lexical retrieval in bilingual individuals [37, 38] and that has items that are generally frequent in both English and Spanish cultures. Additionally, we compared confrontation naming on these two tasks with category generation across three categories for the reasons described above. In addition to examining accuracy on the confrontation naming task, we also systematically examined the nature of target and nontarget language errors that were produced by patients and controls. Likewise, in addition to examining the number of correct words generated on the category generation task, we also examined strategies in verbal fluency including semantic clusters and switches between subclusters across three semantic categories.

In addition to comparing the three lexical access tasks across two languages (English, Spanish), the main goal of this paper was to examine the effect of language proficiency on differences in bilingual lexical access in normal bilingual controls as well as in individuals with bilingual aphasia. To this end, we obtained detailed measures of language background, use, and proficiency in both bilingual controls and in patients with bilingual aphasia. We predicted that bilingual controls would outperform the patients on all three measures of lexical access, but both groups would demonstrate a variance in the nature of strategies employed in lexical retrieval. As such, we expected bilingual controls to produce different semantic clusters and switches and fewer semantic errors compared to bilingual individuals with aphasia. In addition, we predicted language proficiency measures such as language exposure, self-rating of language proficiency, and other parameters to positively correlate with the extent to which participants successfully retrieved words in the two languages.

## 2. Methods

### 2.1. Participants

Twelve Spanish-English bilingual nonbrain damaged individuals between the ages of 18 and 70 (mean age = 34.92 years, standard deviation = 18.89, see Table 1 for a complete description of demographic information). Control subjects were paid $10 each for their participation. Ten Spanish/English bilingual speakers with aphasia participated in the study (see Table 2 for a complete description of demographic information). All participants experienced a single, unilateral cerebral vascular event (CVA, or stroke) in the distribution of the left middle cerebral artery at least 6 months prior to initiation of the experiment with the exception of BA04 who experienced a gunshot wound in the left hemisphere. Participants with apraxia were excluded from the study because the motor complexity can impact oral naming, which was the main task in the study.

#### 2.1.1. Assessment of Language Proficiency Levels

All participants received extensive background language assessments and a comprehensive LUQ [39]. This questionnaire obtained information about the period of* age of language acquisition* (AoA). Next, participants were required to* self-rate their proficiency* (prestroke for bilinguals with aphasia) in each language in terms of their ability to speak and understand the language in formal and informal situations and read and write in each language. Again, an average proportion score in each language reflected participants' perception of their own* language ability rating* (LAR). Additionally, a proportion of language exposure in hearing, speaking, and reading domains during the entire lifetime for each individual was obtained. A weighted average of the proportion of language exposure in the three domains was obtained for each language; for the participants, this information primarily reflected their prestroke* lifetime language exposure*. A similar set of questions obtained a proportion of confidence in hearing, speaking, and reading domains during the entire lifetime for each individual. A weighted average of the proportion of confidence in language use in the three domains was obtained for each language; for the participants, this piece of information primarily reflected their prestroke* language confidence use*. Participants estimated the time spent conversing in each language hour by hour during a typical weekday and typical weekend. A weighted average of this score reflected the proportion of language use in the two languages; for the participants with aphasia this piece of information reflected their* current (poststroke) language* use. Participants were also asked to rate their* family proficiency* (estimates of parent/sibling proficiency) in each of the two languages. Finally, participants also filled out a detailed* educational history* form in which they were asked to provide the language of instruction and the predominant language used during educational interactions.

#### 2.1.2. Assessment of Language Impairment for Participants with Aphasia

Because there is inadequate evidence to guide* a priori* hypotheses about lexical-semantic impairments, no explicit criteria other than the ability to perform the experimental task were set for inclusion in the experiment. The three pictures subtest of* Pyramids and Palm Trees (PAPT)* [20], the* Bilingual Aphasia Test (BAT)* [40], and the* Boston Naming Test *[12, 41] were administered in both languages (English/Spanish) on separate days by separate examiners (see Table 3 for score information). The BNT was administered in its entirety (all sixty items) according to the protocol including the guidelines for basal and ceiling scoring as indicated in the manual. Scoring for the Spanish items was done according to the procedures reviewed by Kohnert et al. [12]. With the exception of the BNT which was analyzed further for differences, results from the remaining tests are reported as patient demographic information to provide additional information about the nature of language impairment.

### 2.2. Materials

In addition to the BNT, a second picture naming task that included primarily high frequency concrete nouns obtained from specific categories (Bilingual Picture Naming Task, BPNT) was administered. Stimuli for this task were chosen from our previous work that included a corpus of 200 words that varied across semantic categories [37, 38]. In both language pairs, cognates (e.g.,* elephant* and* elefante*) and words with at least 50% phonetic similarity (e.g.,* cat* and* gato*) were eliminated from the set. The picture stimuli were chosen from Art Explosion Software (NOVA Inc.) and modified to approximately 4 × 6 inches. The picture naming task consisted of 108 pictures. Stimuli were presented in language blocks with the order of stimuli pseudorandomized within each block to ensure that items from the same category were not presented sequentially. Prior to presentation of stimuli in each language, the bilingual clinician verbally conversed with the participant for a minimum of five minutes (i.e., general everyday conversation) to ensure that participants were aware of the target language and to facilitate lexical access of the target language.

All participants were also administered a Category Generation (CG) task as a measure of verbal fluency. Three categories were selected:* animals, clothing*, and* food *in English and Spanish. Participants were asked to produce as many semantically related words in two minutes in each of the assigned categories. Again, the order of presentation of languages and categories for the task was counterbalanced across sessions for each participant.

### 2.3. Data Scoring

#### 2.3.1. Picture Naming Scoring

For both naming tests, bilingual controls were shown the target stimuli and given up to thirty seconds to generate a response. Responses were counted as correct if they matched the target response. All other responses were coded on a 20-point error scale that included the following error codes: no response; neologism; perseveration; unrelated word; circumlocution; semantic error; mixed error; phonemic error; correct in nontarget language; accent influence in target language (see Table 6 for descriptions and examples). Target language indicates the language in which testing was taking place at the time. Nontarget language denotes responses that were given in the language not being tested.

The same scoring procedure was used for patients and controls, with minor differences made to compensate for the participants' deficits. In particular, responses were counted as correct if they matched the target response, or contained one phonemic substitution, omission, or addition to the target response; however, for controls, responses had to be accurate productions of the target. Additionally, participants with aphasia were given up to one minute to generate a response to the stimuli pictures.

#### 2.3.2. Category Generation Scoring

For the CG task, the responses of all participants were transcribed and tabulated. This was performed separately for each category and each language. Three measures were obtained from this data: (a) the total number words produced, (b) total correct words produced, (c) mean semantic cluster size, and (d) mean semantic switching in each subcategory for each language, Spanish and English [22, 34]. Outlined below is the scoring procedure for the four categories analyzed. 

*(a) Total Words.* The number of responses, either intelligible or unintelligible, was calculated for each category and language. 

*(b) Total Correct Words.* The accuracy of the words produced in the task was determined through a 20-point error analysis procedure outlined in Table 6. Only intelligible and appropriate words for each category and language were deemed correct. Incorrect responses and any cross linguistic errors, perseverations, two or more repetitions of the same item, were considered as incorrect items.

*(c) Mean Semantic Cluster Score.* In order to calculate clusters produced within each category, several constraints were utilized based on previously published work. For the category of* animals*, the method of analysis was taken directly from Tschirren et al. [34]. The coding system for* clothing* was guided by work done by Rosselli et al. [25]. A coding system for* food items* was developed by applying the methods stated in [21]. The average of all of the semantic clusters in one category and one language was then determined for each subject to produce a final score. (The individual categories are listed in Appendix A.) 

*(d) Mean Semantic Switching Score.* The scoring for the mean semantic cluster score was consistent between each category and each language [34]. This score was calculated as the total amount of changes between clusters (Appendix A).

We did not collect formal measures of reliability. The transcription of oral responses was completed by the testing clinician and the error coding was performed by a research assistant who checked all transcribed responses against the targets prior to coding the errors.

## 3. Results

### 3.1. Analysis of Language History and Proficiency

Tables 1 and 2 reveal that there were differences between the two groups in terms of language history and proficiency. Simple factorial ANOVAs were performed on the various variables (e.g., language ability rating, lifetime language exposure) with group (patient, control) and language (English, Spanish) as independent variables. Results showed a significant main effect of group (*F*(1, 42) = 6.9, *P* < 0.01) and language (*F*(1, 42) = 4.3, *P* < 0.05) indicating that language ability ratings were generally higher for the controls relative to the patients (*P* < 0.05) and in Spanish relative to English (*P* < 0.05). For lifetime language exposure, a significant interaction effect of group and language was observed (*F*(1, 42) = 6.8, *P* < 0.01) indicating that lifetime exposure in Spanish was higher than English for patients (*P* < 0.01) but no significant differences were observed for controls. Similarly, for current language use, a significant interaction of group and language was observed (*F*(1, 42) = 25.7,  *P* < 0.0001) indicating that current language use was higher in English than in Spanish (*P* < 0.01) for controls, whereas current language use was higher in Spanish than in English (*P* < 0.001) for patients. Interestingly, current use of Spanish in the patients was higher than controls (*P* < 0.01). Analysis of language confidence revealed a significant effect of language (*F*(1, 42) = 5.7, *P* < 0.02) with the overall confidence in Spanish being higher than in English. Analysis of family proficiency revealed significant main effects of language (*F*(1, 42) = 19.5, *P* < 0.0001) and interaction effects of group and language (*F*(1, 42) = 4.8, *P* < 0.03) essentially indicating higher family proficiency in Spanish relative to English in patients (*P* < 0.0001), however, the differences were not significant for controls. Analysis on education history was not significant for patients or controls. In summary, these results indicate that both groups demonstrated greater language history and proficiency in Spanish than in English, with the difference between the two languages being larger for the patient group than the control group. Notably, controls demonstrated an interesting split between language history (where values were generally higher in Spanish than English) and current language use (where current use was higher in English than in Spanish).

### 3.2. Picture Naming

Separate regression analyses were used to analyze the dependent variables (performance on the BNT and BPNT) to investigate the factors most responsible for the performance of the groups. The categorical predictors were group (patient, controls) and language (English, Spanish), and the continuous predictors were the variables of the LUQ: LAR, Confidence, Lifetime Exposure, Current Exposure, Family Proficiency, and Education History. For BNT, the overall regression equation was significant (*R* ^2^ = 0.834, *F*(1, 38) = 21.14, *P* < 0.00001). The significant predictors were group (*β* = 0.68, *t*(38) = 9.31, *P* < 0.0001), LAR (*β* = 0.29, *t*(38) = 3.01, *P* < 0.001) and language (*β* = 0.25, *t*(38) = 2.74, *P* < 0.01). For the* BPNT*, which was also significant (*R* ^2^ = 0.765, *F*(1, 36) = 13.03, *P* < 0.0001) significant predictors of performance were group (*β* = 0.52, *t*(34) = 5.91, *P* < 0.0001) and LAR (*β* = 0.46, *t*(34) = 4.00, *P* < 0.001).

Since the regression equations revealed group and at least one aspect of language proficiency to be major predictors for both the BNT and BPNT, the data for the patients and bilingual controls were separated andanalyzed to examine if differences in language performance was observed once language proficiency measures were controlled within each participant group. Also, since the regression analysis for both picture naming tasks revealed LAR as the only significant LUQ predictor, only this variable was entered into a subsequent ANCOVA analysis, with language as the independent variable. For the BNT, there was a significant effect of language even after controlling for LAR (*F*(1, 21) = 16.68, *P* < 0.001). Post hoc tests indicated that naming accuracy on the BNT was higher in English than Spanish (*P* < 0.005). For the BPNT, there was also a significant effect of language after controlling for LAR (*F*(1, 21) = 8.87, *P* < 0.05). However, the post hoc analysis was not significant (*P* > 0.20) with trends indicating that naming performance in English was slightly higher than in Spanish. Results for the bilinguals with aphasia were not significant on the ANCOVA analysis for either BNT or BPNT.

### 3.3. Error Analysis

**Figure 1:**
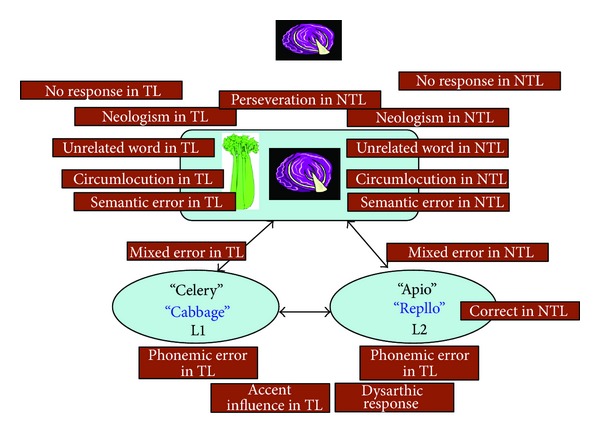
Schematic explaining the hypothesized locus of naming errors that is based on a two-step model of naming that includes semantic access and phonological access in the two languages. All error types may occur in the target language (TL) or nontarget language (NTL). No responses, perseverations, and neologisms are presumed to occur prior to semantic access of the target lemma. Unrelated word, circumlocutions, and semantic errors may occur due to varying degrees of incomplete access at the semantic representation level. Mixed errors (combinations of semantic and phonological errors) may occur due to impaired connections between semantic and language specific phonological levels. Phonemic errors, accent influences, and dysarthric responses are all presumed to occur after language specific phonological access has occurred. Cross-language translations are coded as correct responses in the nontarget language.

**Figure 2:**
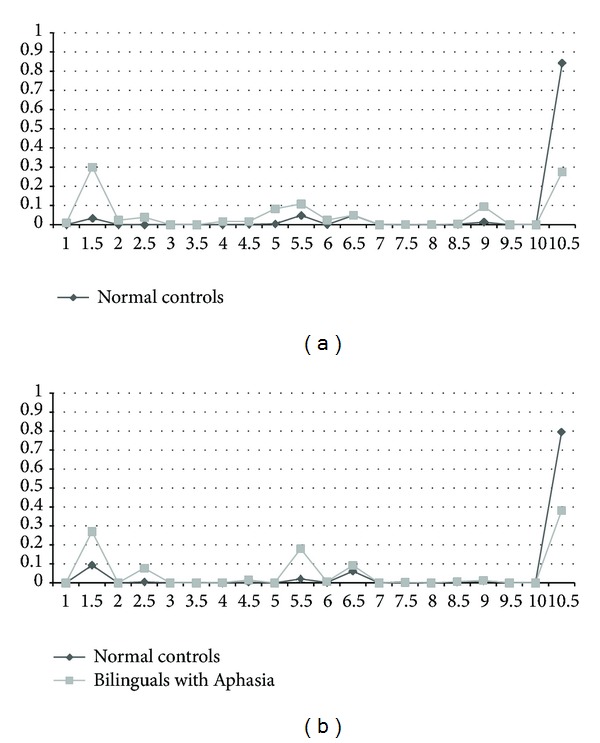
(a) Comparison between accurate production and errors on BPNT for normal controls and bilinguals with aphasia on English targets. (b) Comparison between accurate production and errors on BPNT for normal controls and participants with aphasia on Spanish targets. Correct responses are scored 10.5 (Correct responses in TL). The greatest errors made being No response/idk in TL (1.5), Circumlocution in TL (5.5), Correct response in NTL (9). Error Percentages Spanish (across patients): (1) No response/idk NTL. (1.5) No response/idk TL. (2) Neologism in NTL. (2.5) Neologism in TL. (3) Perseveration to a nonprobe. (3.5) Perseveration to a probe in session. (4) Unrelated word in NTL. (4.5) Unrelated word in TL. (5) Circumlocution in NTL. (5.5) Circumlocution in TL. (6) Semantic error in NTL. (6.5) Semantic error in TL. (7) Mixed error in NTL. (7.5) Mixed error in TL. (8) Phonemic error in NTL. (8.5) Phonemic error in TL. (9) Correct in NTL. (9.5) Dysarthric/apractic intelligible response. (10) Accent Influence in TL. (10.5) Correct in TL.

Responses on the BPNT were further analyzed for the nature of errors produced (providing stimulus cues during BNT makes it difficult to interpret the nature of semantic errors on the task) and interpreted within a framework of lexical access (see Figure 1). Analysis of responses for the BPNT showed that despite the significant differences in accuracy and distribution of error types, no significant differences were observed between bilingual controls and participants with aphasia on English error types (*t*(20) = 0.32; *P* = 0.06). As seen in Figure 2, bilingual controls performed with 84.3% accuracy on English targets. Error types greater than 1% were (a) Circumlocution in target language (4.9%), (b) Semantic error in target language (4.9%), (c) No response/idk in target language (3.8%), and (d) Correct in nontarget language (1.3%). The remaining error types were produced either less than 1% of the time or were not produced at all by bilingual controls in English. Participants with aphasia produced a greater variety of error types, evidenced by their average accuracy of 27.5% in English. The main error types were No response/idk in target language (30%), Correct in nontarget language (9.4%), Circumlocution in nontarget language (10.9%), Neologism in target language (4.8%), Semantic error in target language (3.9%), Neologism in nontarget language (2.4%), Semantic error in nontarget language (2.4%), Unrelated word in nontarget language (2.1%), Unrelated word in target language (1.7%), and Circumlocution in target language (1.1%).

The Spanish data in Figure 2 show even greater similarity between the bilingual controls and participants with aphasia in terms of types of errors produced than the English data (*t*(20) = 0.33, *P* = 0.20). Bilingual controls performed with 79.5% accuracy. Error types greater than 1% included (a) No response/idk in target language (9.3%), (b) Semantic error in target language (6.2%), (c) Circumlocution in target language (2.1%), and (d) Correct in nontarget language (1.07%). Other error types were produced either below 1% or not produced at all by this group. Participants with aphasia performed with 38.1% accuracy in Spanish. The main error types were No response/idk in target language (27%), Circumlocution in target language (17%), Semantic error in target language (9.2%), Neologism in target language (7.8%), Unrelated word in target language (1.5%), and Correct in nontarget language (1.3%).

### 3.4. Category Generation Task

As in the picture naming tasks, a regression analysis was performed on the number of correct words (across the three categories), mean semantic cluster scores, and mean semantic switching scores on the CG task, the categorical predictors were group (patient, bilingual controls) and language (English, Spanish), and the continuous predictors were the variables of the LUQ: LAR, Confidence, Lifetime Exposure, Current Exposure, Family Proficiency, and Education History. 

*(a) Correct Words.* A regression analysis for total correct words was significant (*R* ^2^ = 0.922, *F*(1, 36) = 22.58, *P* = 0.00), the strongest predictor on the task was group (*β* = 0.764, *t*(36) = 10.56, *P* = 0.00), followed by language of the task (*β* = 0.273, *t*(36) = 3.09, *P* < 0.001). Thus, controls produced more words than patients, and words generated in English were higher than in Spanish (*P* < 0.05). Also, of the variables assessed with the LUQ, LAR was the only significant predictor (*β* = 0.226, *t*(36) = 2.65, *P* < 0.01). 

*(b) Mean Semantic Cluster Score.* The regression analysis for the mean semantic cluster scores was significant (*R* ^2^ = 0.753, *F*(1, 36) = 12.89, *P* < 0.0001), and the strongest predictor of performance on the task was group (*β* = 0.677, *t*(36) = 5.30, *P* < 0.0001). Bilingual controls performed significantly higher semantic clusters in both English and Spanish (*P* < 0.05). The only other significant predictor of performance was once again LAR of the LUQ (*β* = 0.222, *t*(36) = 2.06, *P* < 0.05).

*(c) Mean Semantic Switching Score.* The regression analysis for mean semantic switching score for the normal subjects or participants with aphasia did not reveal any significant influence of the LAR on the categorical measures or differences between the measures.  Table 4, however, showed differences between controls and patients, which was confirmed in individual *t*-tests; bilingual controls had a higher semantic switching score in English (*t*(20) = 2.8,   *P* = 0.01) and Spanish (*t*(20) = 4.96, *P* < 0.001) than their patient counterparts.

To further understand patterns of lexical-access within each of the participant groups, data were separated and analyzed. Three ANCOVAs (with LAR as the covariate) were performed for each group for each of the dependent variable (total correct words, mean semantic cluster scores, and mean semantic switching scores). 

**Figure 3:**
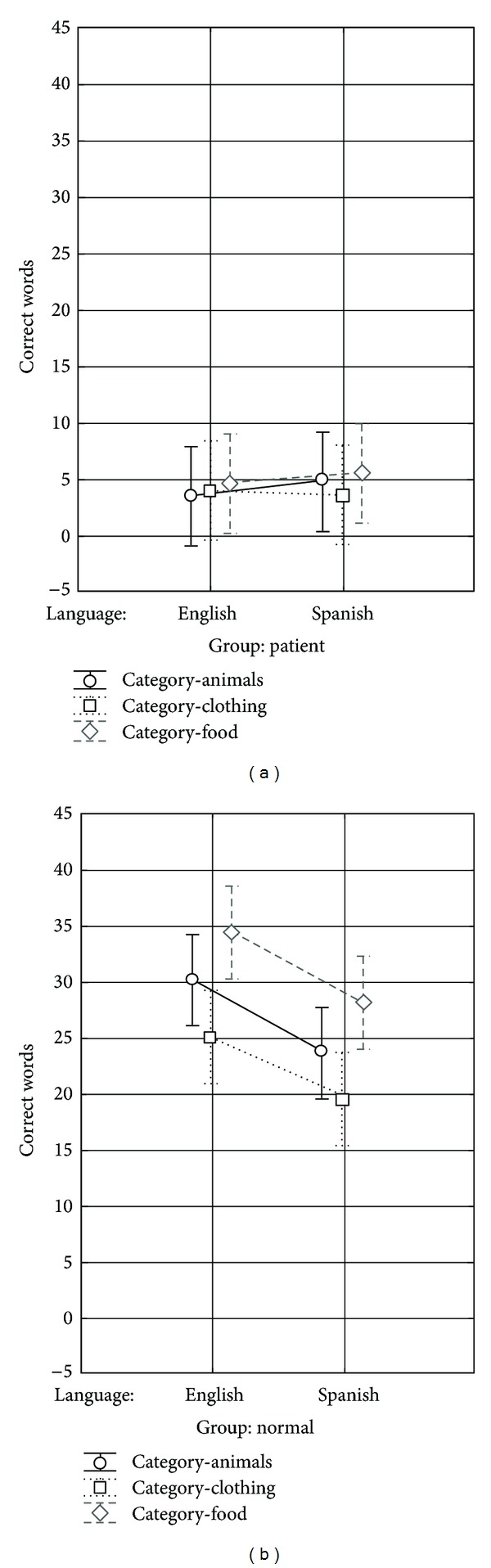
Mean number of correct words for the category generation task for (a) patients with aphasia and (b) normal controls in English and Spanish for three categories: animals, clothing, and food.

*(a) Correct Words.* An ANCOVA for the bilingual control data revealed that LAR did in fact influence the effect of language and category on the correct words. Firstly, there was a significant main effect of language (*F*(1, 71) = 32.8,   *P* < 0.000001) and a main effect of category (*F*(2, 71) = 11.8,   *P* < 0.00005) after controlling for the LAR. Post hoc tests indicated that, for language, the total correct words were significantly greater in English than Spanish (*P* < 0.0001). For category, the total correct words for* food* items differed significantly from the* clothing* items (*P* < 0.00005) and the total correct words for* animals* differed significantly from* clothing* (*P* < 0.05). The ANCOVA was not significant for the participants with aphasia (Figure 3).

*(b) Mean Semantic Cluster Score.* A significant main effect of language was seen on the mean semantic cluster score on the ANCOVA (*F*(1, 71) = 10.2,   *P* < 0.005) and the main effect of category was also significant (*F*(2, 71) = 3.32, *P* < 0.05). The post hoc tests for the mean semantic cluster score analysis revealed that, for language, the mean semantic cluster scores in English were significantly more than Spanish (*P* < 0.01). Additionally, for the categories, the mean semantic cluster scores for the* food *items were significantly higher than for the* clothing* items (*P* < 0.05). The categories of* food* items and* animals* did not show any significant difference. The ANCOVA for participants with aphasia data was not significant.

*(c) Mean Semantic Switching Score.* The final ANCOVA analysis on the mean semantic switching score for the normal controls or participants with aphasia did not reveal any significant influence of the LAR on the categorical measures or differences between the measures.

### 3.5. Individual Patient Analysis

**Figure 4:**
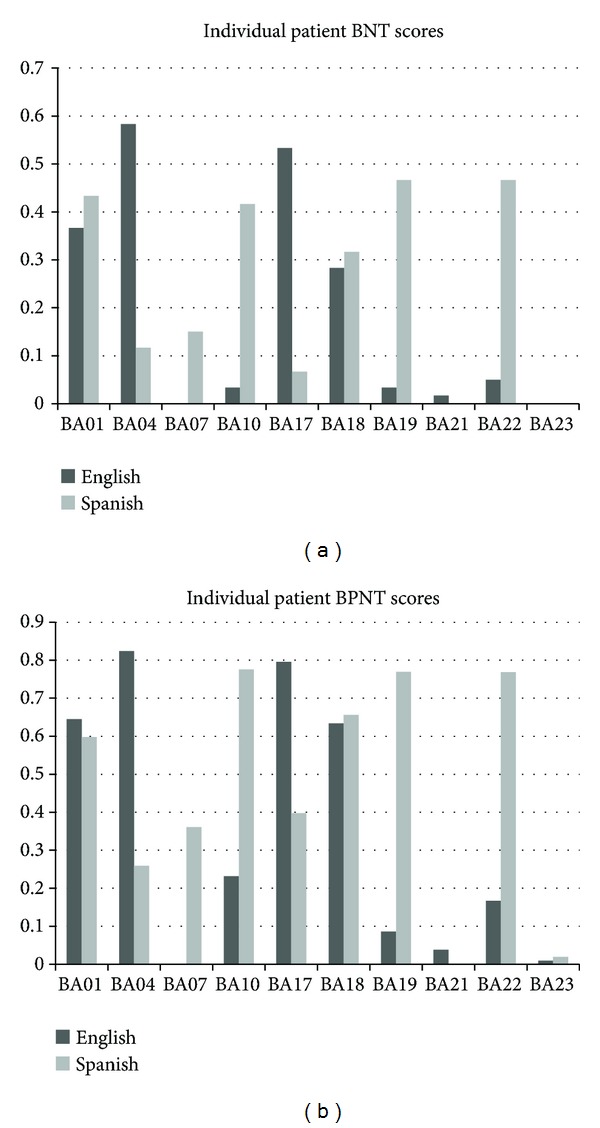
Individual patient accuracy on the two naming tasks: BNT (a) and BPNT (b) in English and Spanish.

**Figure 5:**
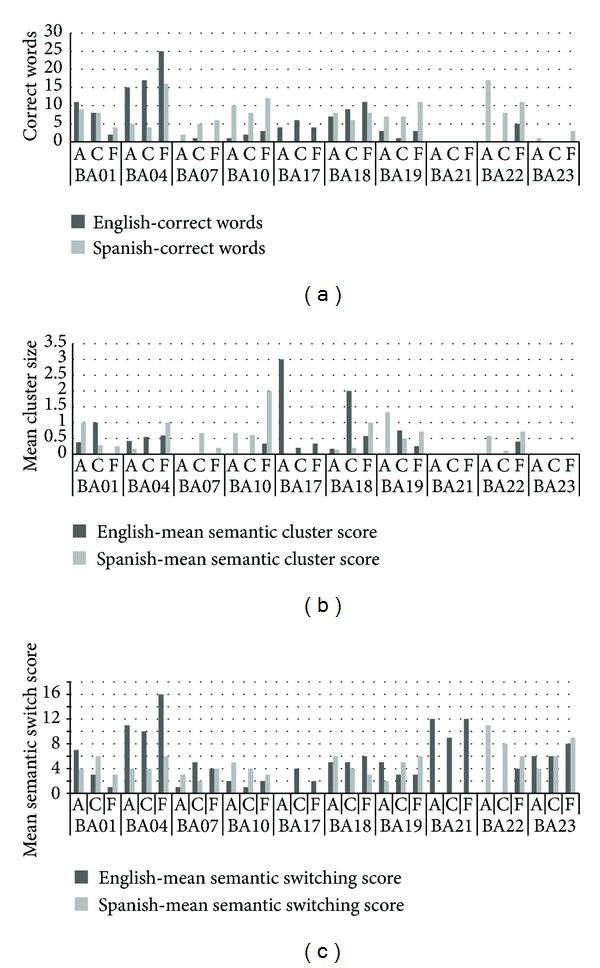
Results of category generation task for individual patients across three categories Animals (A), Food (F), and Clothing (C) in English and Spanish. (a) Correct Word Scores for the Category Generation Task in English and Spanish across each participant in the categories, (b) Mean Semantic Cluster Scores, and (c) Mean Semantic Switching Scores. Comparing each participant in their correct responses provided in English and Spanish exemplifies their dominant languages and individual differences.

Because the parametric statistical analysis for the patients was mostly nonsignificant, a more qualitative inspection of the data was carried out. As is evident in Figures 4 and 5 the results of the participants with aphasia showed more variation than did those of the normal controls on all three tasks (BNT, BPNT, and CG task). Individual inspection of the participant data showed that participants BA04 and BA17 produced more correct responses in English than Spanish across the three tasks. On the other hand, participants BA07, BA10, BA19, BA22, and BA23 produced more correct responses in Spanish than English in all three tasks. Two patients, BA01 and BA18, received scores that were remarkably similar in both languages, while participant BA21 produced either no correct responses or performed with very low accuracy in both languages, for all tasks. With regards to the nature of category-specific access on the category generation task, the broad variety of responses and scores were independent of category; however, it was clear that the categories* Animals *and* Food* were easier to access than* Clothing *for most patients, a finding that was similar to the control data. Also, only two of the ten patients showed language differences in their semantic clustering ability, with BA17 producing more clusters in English and BA10 producing more clusters in Spanish. Likewise, only a few patients (BA04, BA17, and BA22) showed language-specific differences in their semantic switching scores, while other patients demonstrated similar switching patterns in English and Spanish.

### 3.6. Across Task Correlations

Recall that, in the introduction, we argued that the three word retrieval tasks assessed similar aspects of lexical access, but the nature of the tasks placed slightly different demands on lexical access. In the final analysis, we systematically correlated the three tasks administered with the only significant continuous predictor in the regression analysis, LAR to examine to what extent these measures actually correlated with each other. Bilingual controls and bilingual individuals with aphasia were separated for this analysis again to prevent group-driven effects in the results. The bivariate correlation analysis revealed for the bilingual controls, significant (*P* < 0.05) correlations emerged between LAR and correct words generated, LAR and BNT, and LAR and BPNT in English (see Table 5). Additionally, significant correlations were observed between BPNT and BNT, and correct words generated and BPNT (and BNT) responses. In Spanish, significant (*P* < 0.05) correlations emerged between correct words generated and BNT, BNT and BPNT, LAR and BNT, and LAR and BPNT. For bilingual individuals with aphasia, in English significant (*P* < 0.05) correlations emerged between correct words and BPNT, LAR, and correct words, LAR, and BNT, and LAR and BPNT. In Spanish, significant (*P* < 0.05) correlations emerged only between correct words generated and BPNT.

## 4. Discussion

The present study examined the nature of lexical-access in normal bilinguals and in participants with bilingual aphasia across three different lexical-semantic access tasks (BNT picture naming, BNPT picture naming, and verbal fluency). Results are discussed in the context of the goals proposed in the study.

### 4.1. Comparison of the Three Lexical Retrieval Tasks

The results from the three lexical retrieval tasks revealed several similarities and some important differences. Notably, the results from the two confrontation naming tests, the BNT and BPNT, were somewhat different regarding the factors that drove performance for each test. For the BPNT, Group, LAR, Confidence, and Family Proficiency were significant determiners of performance. However, for the BNT, only Group, LAR, and Language were significant determiners of performance.

As previously mentioned, the BPNT included two sets of high frequency words in English and Spanish. Many of the items on the BNT, however, are low frequency words in spontaneous speech (e.g.,* abacus*) and are not translated particularly well in Spanish. Indeed previous studies that have examined BNT in Spanish and English in normal bilinguals have described lower performance accuracy [13] in Spanish. After comparing two groups of bilingual adults (Spanish/English and French/English) and monolingual English adults on the BNT, it was determined that, for both bilingual groups, mean test scores were significantly below the monolingual group while not significantly differing from each other. The study suggests variability between each bilingual group and individual participants, with less significance derived from background influences. Consequently, one would expect performance in the dominant language in bilinguals to be far greater than performance in the nondominant language for the BNT, while differences between the two languages on the BPNT would be less great due to the high frequency of the items in both languages, which was one of the findings of the study.

With respect to the category generation task, results indicated that the ability to semantically cluster, switch, and efficiently produce correct words in the task was influenced by Group, Language, and LAR. Previous studies assessing the performance of bilingual Spanish/English and monolingual English and Spanish speakers additionally demonstrated a significantly greater performance of bilingual participants in verbal fluency tasks depending on the age of acquisition and level of bilingualism without, however, an effect of language [23, 25]. Differences may have arisen between the above two studies and the data presented here based on the geographic sampling of patients and level of balanced bilingualism found within our groups (see further on individual patient analysis). What our results indicate is that, across the three tasks, when language proficiency self-rating was controlled for, at least for the controls, performance in English was higher than performance in Spanish. These results are underscored by the fairly robust correlations between the three lexical retrieval tasks and their overall correlation with LAR.

### 4.2. Performance Differences between Languages

Overall, the data revealed that the normal controls were more accurate in English than in Spanish on the BNT, BPNT, and both correct words and mean semantic cluster scores on the category generation task, even when language proficiency was taken into account. In contrast, for aphasic participants, there was no significant effect across languages. This observation is interesting against the comparison of the analyses of language use and background for both groups. While both groups demonstrated greater language exposure and proficiency in Spanish than in English, the difference between the two languages was larger for the patient group than the control group. Notably, controls demonstrated an interesting split between language history (where values were generally higher in Spanish than English) and current language use (where current use was higher in English than in Spanish). Since the current lexical retrieval tasks tap into real-time lexical access, perhaps current language use may be reflective of the degree of lexical access. For patients, the overall group analysis were not significant; however, individual analyses showed that there were more patients with higher performance in Spanish than in English.

Results from the three categories,* animals*,* food*, and* clothing* on the category generation task revealed that the differences between* food* and* clothing* items for the total correct words and mean semantic cluster score and the differences between the* animals* and* clothing* items for the total correct words also remained after controlling for LAR for the controls (and to a lesser extent for some of the patients). Therefore, the differences in performance observed for the normal controls between each category had a large cultural influence and were based on the individual's own vocabulary and lifetime experiences [23]. In contrast, Pekkala et al. [27] showed that, between two normal monolingual groups of Finnish and English-speaking subjects, differences in performance on semantic verbal fluency tasks were minimal even after normalizing for educational influences. They, therefore, suggested that cultural and language differences do not have a significant contribution to performance in monolingual normal controls. As an alternative explanation, the normal controls, in general, possessed a much greater ability of producing sequential clusters of words and the ability to switch between clusters in all categories that were tested, which was assumed to be a function of their greater level of cognition and effective semantic strategizing techniques [21].

### 4.3. Differences across Participant Groups

As would be expected, normal controls were significantly better at lexical retrieval on all three tasks relative to bilingual patients with aphasia. At first glance this difference between the groups may suggest that patients and normal controls perform radically differently on the picture naming tests. However, Figure 2 shows that both groups produce similar errors in both languages, with the difference being the rate of each error type between the groups. This finding suggests that despite lexical retrieval deficits associated with stroke, the basic mechanism and potential breakdown of lexical retrieval in participants with aphasia on naming tasks are no different from that of the normal controls (Figure 1). For instance, both patients and controls produced mainly semantic paraphasias and circumlocutions in the target language/nontarget language. Consistent with our findings, in a study examining the nature of semantic errors in monolingual aphasia, Dell et al. [42] found that individuals with and without aphasia performed similarly with respect to error type and that semantic paraphasias produced by aphasic individuals are a continuation of semantic substitution errors in nonaphasic speech.

With respect to the category generation task, even though bilingual controls produce many more items than bilinguals with aphasia, the differences between the two groups are smaller for the semantic cluster scores, contrary to the initial predictions of the study (Table 4). This suggests that the strategies for clustering may not be all that different for the two groups. Troyer et al. [18] found that while clustering and switching were correlated with performance on verbal fluency, there was a greater effect of switching on phonemic fluency. Although a negative correlation between semantic clustering and switching was found in the Troyer study, optimal performance requires a balance between a decrease in the number of switches and the total number of words produced. In summary, these results suggest that while bilingual individuals with aphasia may not be able to access an item successfully, they appear to cluster their responses within appropriate semantic subcontexts. Finally, while patients with aphasia produced fewer semantic switches than their controls, the ratio of correct words to semantic switches was not all that different between patients and controls (Table 4).

### 4.4. Individual Patient Performance

In general, the low overall accuracy of the aphasic participant group precluded the possibility of drawing conclusions about the effect of brain damage once prestroke language proficiency was controlled. For all three tasks, it was observed that there were large individual differences creating much variation in the data to interpret. Observations of the results for BA04 and BA17 for the BNT and BPNT are especially noteworthy. Despite reporting Spanish as the L1 and near equal amounts of time speaking each language, BA04 and BA17 performed with greater accuracy in English than in Spanish on both the BNT and the BPNT. Other patients' naming accuracies were commensurate with their premorbid relative dominance in each language.

Similarly on the CG task, closer inspection of the results for individual participants revealed that participants with aphasia, like their controls, produced items within each category and each language, reflective of their relative dominance in each language. While a few participants reported they were English dominant (BA04, BA17, and BA21), only BA04 and BA17 produced more items in English. Other participants who were Spanish dominant (BA07, BA10, BA19, BA22, and BA23) produced more items in Spanish. There were also participants who showed no differences between the outputs in the two languages. These results underscore the influence of premorbid language proficiency on lexical retrieval even after brain damage and provide some validation for our reported measures of language use, exposure, and proficiency.

### 4.5. Influence of Language Proficiency on Lexical Retrieval

Interestingly, the initial regression analyses showed that, of all the LUQ variables, only LAR was consistently a significant predictor of performance across all five measures of lexical access (across the three tasks). This effect is due to nature of the variable: LAR is a compound, albeit subjective, judgment comprised of all the other variables of the LUQ. It therefore represents all the other variables of the LUQ combined. The results of the regression suggest that each factor of the LUQ does predict performance on the lexical retrieval tasks examined, but only when they are combined do the individual factors become significant as performance predictors. Of note is the difference between current language use and all other measures of the language exposure and proficiency for the bilingual controls. These results validate the need to obtain a multidimensional view of language use and exposure, and possibly the LAR captures some of that multidimensionality as it is a measure of the participants own judgment of their proficiency.

Importantly, LAR-English correlated with naming accuracy on BNT-English, BPNT-English, and correctly generated words in English for both the bilingual controls and bilingual patients with aphasia. Correlations between LAR-Spanish were less robust for both the controls and patients, perhaps indicating the lack of stability of this measure in obtaining a comprehensive lifespan history of Spanish language usage, of notable concern since all the patients (and several controls) were native Spanish speakers. Nonetheless, the observation that different measures of lexical retrieval correlated with a compound measure of language proficiency is an encouraging preliminary observation. The results of Kohnert et al. [12] and the present study underline the importance of independent self-reported measures of language proficiency in assessing language impairment of bilingual individuals with aphasia. While much work needs to be done in terms of delineating specific aspects of language proficiency (life time exposure, family proficiency, or education) that differentially influence various language processing tasks, the present study demonstrates that, until then, a composite albeit subjective measure of self-rated language ability is a good place to start.

## 5. Conclusion

The large differences in performance of the normal subjects and bilingual participants with aphasia demonstrate a fundamental lexical retrieval deficit in bilingual individuals with aphasia, but one that is further influenced by language proficiency in the two languages. The findings of our study indicate that normal controls and participants with aphasia make similar types of errors in both English and Spanish and develop similar clustering strategies despite significant performance differences between the groups.

## Figures and Tables

**Table 1:** Demographic information for bilingual normal controls.

Control	Age	Sex	AoA		Lifetime exposure %		Confidence %		Current exposure %		Family proficiency %		Education history %		Language ability rating %	
			Eng.	Sp.	Eng.	Sp.	Eng.	Sp.	Eng.	Sp.	Eng.	Sp.	Eng.	Sp.	Eng.	Sp.
BC01	18	F	5	0	40.83	59.17	48.33	75.83	79.35	20.65	58.33	91.67	55.56	44.44	97.14	85.71
BC02	19	F	8	0	53.89	46.11	87.50	98.61	93.33	6.67	58.33	91.67	61.11	38.89	100.00	91.43
BC03	21	F	0	9	82.94	17.06	100.00	31.25	58.14	41.86	100.00	33.33	72.22	27.78	100.00	91.43
BC05	42	M	0	0	54.63	45.37	88.10	100.00	75.21	24.79	91.67	91.67	11.11	88.89	100.00	100.00
BC06	20	F	2	0	57.34	42.66	88.33	89.44	63.79	36.21	75.00	100.00	72.22	27.78	100.00	100.00
BC07	60	M	30	0	45.13	54.87	55.00	100.00	88.95	11.05	8.33	100.00	33.33	66.67	62.86	100.00
BC08	20	M	0	0	38.10	61.90	95.56	95.56	50.00	50.00	91.67	100.00	50.00	50.00	80.00	100.00
BC09	20	F	3	0	50.00	50.00	86.94	85.56	57.50	42.50	37.50	100.00	72.22	27.78	100.00	94.29
BC10	58	F	48	0	20.46	79.20	24.14	75.86	45.58	54.42	0.00	100.00	0.00	100.00	48.57	100.00
BC11	34	F	14	0	88.07	11.93	100.00	40.63	91.07	8.93	100.00	0.00	100.00	0.00	100.00	71.43
BC12	62	M	12	0	42.47	57.53	61.54	100.00	82.77	17.23	50.00	100.00	16.67	83.33	94.29	100.00
BC14	61	F	10	0	43.90	56.10	37.91	100.00	40.91	59.09	0.00	100.00	25.00	75.00	45.71	100.00

Note: AoA: Age of acquisition, Eng.: English, Sp.: Spanish.

**Table 2:** Demographic information for bilingual individuals with aphasia.

Patient	Age	Sex	AoA		Lifetime exposure %		Confidence %		Current exposure %		Family proficiency %		Education history %		Language ability rating %	
			Eng.	Sp.	Eng.	Sp.	Eng.	Sp.	Eng.	Sp.	Eng.	Sp.	Eng.	Sp.	Eng.	Sp.
BA01	43	M	19	0	28.00	72.00	42.00	94.00	22.00	78.00	33.00	100.00	0.00	100.00	89.00	89.00
BA04	36	M	0	0	74.00	26.00	81.00	100.00	66.00	34.00	67.00	100.00	100.00	0.00	100.00	89.00
BA07	65	F	45	0	10.00	90.00	5.00	100.00	2.00	98.00	0.00	100.00	0.00	100.00	32.00	100.00
BA10	76	M	40	0	4.00	96.00	15.00	100.00	0.00	100.00	0.00	100.00	0.00	100.00	47.00	100.00
BA17	53	M	6	0	66.00	34.00	96.00	98.00	55.00	45.00	75.00	100.00	58.00	42.00	100.00	100.00
BA18	73	F	17	0	40.00	60.00	80.00	100.00	0.00	100.00	58.00	100.00	25.00	75.00	100.00	100.00
BA19	75	M	27	0	16.00	84.00	13.00	76.00	15.00	85.00	0.00	100.00	0.00	100.00	20.00	100.00
BA21	88	F	5	0	72.00	28.00	100.00	100.00	99.00	1.00	100.00	100.00	100.00	0.00	31.00	23.00
BA22	42	M	18	0	10.00	90.00	11.00	92.00	38.00	63.00	17.00	100.00	0.00	100.00	34.00	94.00
BA23	42	F	9	0	33.00	67.00	42.00	100.00	29.00	71.00	33.00	100.00	22.00	78.00	66.00	94.00

Note: AoA: Age of acquisition, Eng: English, Sp: Spanish.

**Table 3:** Average scores for bilinguals with aphasia and bilingual normal controls on BAT-Comprehension, BAT-Semantics, BNT, and BPNT in English and Spanish. Scores for bilingual normal controls are provided for BNT and BPNT (standard deviations are in parenthesis).

Group	BAT Comp %		BAT Sem %		Boston naming test %		Bilingual picture naming task %	
	English	Spanish	English	Spanish	English	Spanish	English	Spanish
Controls					75.00 (21.66)	61.81 (15.33)	85.52 (19.45)	80.73 (12.41)
Patients	47.96 (28.33)	69.26 (20.72)	45.71 (14.90)	51.67 (13.54)	18.83 (22.91)	24.51 (19.44)	34.73 (34.72)	46.05 (29.95)

**Table 4:** Mean correct words on the category generation task, mean semantic cluster scores, and mean semantic switching scores for bilingual normal controls and bilinguals with aphasia (standard deviations are in parenthesis).

Group	Correct words		Mean semantic cluster score		Mean semantic switching score		Mean ratio of correct words to semantic switches	
	English	Spanish	English	Spanish	English	Spanish	English	Spanish
Controls	29.70 (9.10)	24.36 (6.18)	2.07 (0.86)	1.47 (0.50)	8.94 (2.53)	9.75 (2.89)	3.36 (0.81)	2.55 (0.41)
Patients	4.60 (5.90)	5.87 (4.24)	0.36 (0.41)	0.41 (0.36)	5.10 (3.84)	3.93 (2.54)	1.06 (0.84)	1.58 (0.65)

**Table 5:** Pearson correlations of BNT, BPNT, correct words on the category generation task, and LAR administered for bilingual controls and individuals with aphasia. Correlations significant at *P* < 0.05 are highlighted with an asterisk.

Group	Variable	Correct Words-E	Correct Words-S	LAR-E	LAR-S	BPNT-E	BPNT-S	BNT-E	BNT-S
Bilingual controls	Correct Words-E	1	0.277	0.892*	−0.506	0.769*	−0.385	0.818*	−0.211
	Correct Words-S		1	0.270	0.382	−0.137	0.555	−0.003	0.612*
	LAR-E			1	−0.440	0.737*	−0.207	0.787*	−0.147
	LAR-S				1	−0.337	0.846*	−0.364	0.820*
	BPNT-E					1	−0.311	0.961*	−0.118
	BPNT-S						1	−0.281	0.855*
	BNT-E							1	−0.097
	BNT-S								1

Bilingual individuals with aphasia	Correct Words-E	1	0.286	0.707*	−0.151	0.799*	0.027	0.045	−0.229
	Correct Words-S		1	0.013	0.347	0.051	0.796*	−0.386	0.254
	LAR-E			1	0.310	0.878*	0.049	0.636*	−0.183
	LAR-S				1	0.058	0.625	0.325	0.494
	BPNT-E					1	0.132	0.490	−0.428
	BPNT-S						1	−0.070	0.186
	BNT-E							1	−0.122
	BNT-S								1

**Table 6:** Description of error types and examples of errors produced in English and Spanish.

Error	Description	Example	
		English target	Spanish target
No response in nontarget language (1)	No response or response of “I don't know” in the language not being tested	Target: cabbage Response: No me recuerdo	Target: lechuga Response: I don't remember
No response in target language (1.5)	No response/response of “I don't know” in the language in session being tested	Target: glove Response: I don't know	Target: media Response: No sé
Neologism in nontarget language (2)	Unrecognized word in any dialect of the language not being tested after correcting for possible phonemic errors	Target: Counter Response: clov	Target: tiburón Response: babberi
Neologism in target language (2.5)	Unrecognized word in any dialect of the language being tested after correcting for possible phonemic errors	Target: shelf Response: crademan	Target: rastrillo Response: serame
Perseveration to a nonprobe (3)	Repetition at least three times of a neologism or word unrelated to the target and not previously presented to the subject	Target: arm Response: go go	Target: brazo Response: go go
Perseveration to a probe in session (3.5)	Repetition at least three times (in any language) of a word previously presented to the subject but unrelated to the target	Target: necklace Response: baseball (if generated at least twice before)	Target: brazo Response: ring ring (if generated at least twice before)
Unrelated word in nontarget language (4)	Word semantically and phonologically unrelated to the target word in the language not being tested	Target: counter Response: perro	Target: puerta Response: berry
Unrelated word in target language (4.5)	Word semantically and phonologically unrelated to the target word in the language being tested	Target: hook Response: coach	Target: jarra Response: ardilla
Circumlocution in nontarget language (5)	Utterance (description) providing semantic information about the target in the language not being tested	Target: hamburger Response: algo que se come	Target: oso Response: an animal
Circumlocution in target language (5.5)	Utterance (description) providing semantic information about the target in the language being tested	Target: building Response: a structure	Target: perica Response: un para hombre
Semantic error in nontarget language (6)	Semantic substitution/paraphasia in the language not being tested	Target: mop Respose: rastrillo	Target: anillo Response: diamond
Semantic error in target language (6.5)	Semantic substitution/paraphasia in the language being tested	Target: pitcher Response: coffee pot	Target: brazo Response: mano
Mixed error in nontarget language (7)	Combination of two or more errors from analysis criteria in the language not being tested	Target: sword Response: fecha	Target: mapache Response: racooco
Mixed error in target language (7.5)	Combination of two or more errors from analysis criteria in the language being tested	Target: leg Response: musolos	Target: hormiga Response: arinas
Phonemic error in nontarget language (8)	Greater than one phonemic substitution or omission in the language not being tested	Target: robe Response: bete	Target: edificio Response: build
Phonemic error in target language (8.5)	Greater than one phonemic substitution or omission in the language being tested	Target: celery Response: cerelec	Target: aspiradora Response: astirador
Correct in nontarget language (9)	Correct response (including single phoneme substitutions) in the language not being tested	Target: shelf Response: estante	Target: taburete Response: stool
Dysarthric/apractic intelligible response (9.5)	Response from a patient with known dysarthria or apraxia		
Accent influence in target language (10)	Correct response in target language but containing the phonology of the language not being tested	Target: duck Response: dok ([dog])	Target: pollo Response: polo ([powlo])
Correct in target language (10.5)	Correct response (including single phoneme substitution, addition or omission for aphasic participants only) in the language being tested	Target: giraffe Response: giraffe	Target: avestruz Response: avestruzo

## Appendix

### A. Categorization of Items on the Category Generation Task

*Animals*

1. *Living Environment *
  - Africa
    - aardvark, antelope, buffalo, camel, chameleon, cheetah, chimpanzee, cobra, eland, elephant, gazelle, giraffe, gnu, gorilla, hippopotamus, hyena, impala, jackal, lemur, leopard, lion, manatee, mongoose, monkey, ostrich, panther, rhinoceros, tiger, wildebeest, warthog, zebra;
  - Australia
    - emu, kangaroo, kiwi, opossum, platypus, Tasmanian devil, wallaby, wombat
  - Arctic/Far North
    - auk, caribou, musk ox, penguin, polar bear, reindeer, seal;
  - Farm
    - chicken, cow, donkey, ferret, goat, horse, mule, pig, sheep, turkey;
  - North America
    - badger, bear, beaver, bobcat, caribou, chipmunk, cougar, deer, elk, fox, moose, mountain lion, puma, rabbit, raccoon, skunk, squirrel, wolf;
  - Water
    - Alligator, auk, beaver, crocodile, dolphin, fish, frog, lobster, manatee, muskrat, newt, octopus, otter, oyster, penguin, platypus, salamander, sea lion, seal, shark, toad, turtle, whale;
2. *Human Use*
  - Beasts of Burden
    - Camel, donkey, horse, llama, ox
  - Fur
    - Beaver, chinchilla, fox, mink, rabbit
  - Pets
    - budgie, canary, cat, dog, gerbil, golden retriever, guinea pig, hamster, parrot, rabbit
3. *Zoological Categories*
  - Bird
    - budgie, condor, eagle, finch, kiwi, macaw, parrot, parakeet, pelican, penguin, robin, toucan, woodpecker;
  - Bovine
    - bison, buffalo, cow, musk ox, yak;
  - Canine
    - coyote, dog, fox, hyena, jackal, wolf;
  - Deer
    - antelope, caribou, eland, elk, gazelle, gnu, impala, moose, reindeer, wildebeest;
  - Feline
    - bobcat, cat, cheetah, cougar, jaguar, leopard, lion, lynx, mountain lion, ocelot, panther, puma, tiger;
  - Fish
    - bass, guppy, salmon, trout;
  - Insect
    - ant, beetle, cockroach, flea, fly, praying mantis;
  - Insectivores
    - aardvark, anteater, hedgehog, mole, shrew;
  - Primate:
    - ape, baboon, chimpanzee, gibbon, gorilla, human, lemur, marmoset, monkey, orangutan, shrew;
  - Rabbit
    - coney, hare, pika, rabbit;
  - Reptile/Amphibian
    - alligator, chameleon, crocodile, frog, gecko, iguana, lizard, newt, salamander, snake, toad, tortoise, turtle;
  - Rodent
    - beaver, chinchilla, chipmunk, gerbil, gopher, groundhog, guinea pig, hamster, hedgehog, marmot, mole, mouse, muskrat, porcupine, rat, squirrel, woodchuck;
  - Weasel
    - badger, ferret, marten, mink, mongoose, otter, polecat, skunk.

The scoring system is outlined below. The only constraint utilized was for subordinate examples of a particular item. In this case, items were considered to be correct if they had distinct functions (e.g., long sleeve shirt versus short sleeve shirt) or were different species of an animal (pilgrim hawk versus red hawk).

An example of this procedure is from the pretesting task from BA01. This set of words would be grouped successively giving the following scores:

bee
dog
raccoon
ant
raccoon
raccoon
cat
rabbit
horse
bunny
raccoon.

Firstly,* bee* would be given a score of 0 because it is not semantically related to dog in any way. In the same way,* dog*,* raccoon*, and* ant* are all not semantically related, so they would each receive a score of 0. As repetitions are counted, the next two words produced,* raccoon* and* raccoon*, are semantically related (as they are the same word), so they would receive a score of 1. Of the remaining five words,* cat*,* rabbit*,* horse*,* bunny*, and* raccoon*, the first fourare semantically related giving a score of 4, as* cat*,* rabbit*,* horse*, and* bunny* are pets and* bunny *and* raccoon* are animals from North America.

The mean of these scores is then taken to determine an average score for each category:

(A.1) $$\begin{array}{c} \frac{5}{6}=0.833. \end{array}$$

This same procedure is repeated for the posttesting task, and the two values from pre- and posttesting are compared with a basic bar graph.

The semantic switching score for the example above would be 5 (5 arrows above). Again, the scores are calculated for pre- and posttesting and a bar graph is created to compare the values.

*Clothing Items.* The scoring system is still the same for the semantic cluster and semantic switching score as above. The subcategories for clothing are as follows:

1. *similar weather conditions*
  - clothing for each season
    - winter (jacket, sweater, hat, etc.)
    - summer (shorts, bathing suit, sunglasses, etc.);
2. *upper body versus lower body*
  - upper Body
    - shirt, sweater, coat, vest, and so forth;
  - lower body
    - pants, shorts, capris, shoes, and so forth;
3. *accessories*
  - accessories are matched to their appropriate category in the above two subcategories
    - sunglasses, cap, to summer clothing
    - hat, scarf, gloves, mittens, to winter clothing
    - necklace, earrings, rings, tie to upper body clothing;
4. *sets of matching clothing (strong pairs)*
  - pairs of clothes that are usually worn together
    - coat and tie; sweatshirt, and sweatpants, jeans and, t-shirt, socks and shoes, and so forth;
  - different occasions
    - formal wear
      - suit, dress shirt, blouse, tuxedo, and so forth.

*Food Items.* The scoring system is the same as stated in the previous two categories. The subcategories have been grouped based on the following criteria:

1. * beans*
2. * beverages*
  - water, soda, juice, milk, and so forth
3. * breads*
4. * candy*
5. * cold cereals*
6. * condiments*
7. * desserts*
8. * fish*
9. * fruits*
10. * grains/cereals*
11. * junk food*
12. *meats*
  - cold cuts
  - poultry
13. *dairy products*
14. *nuts/seeds*
15. *prepared foods and meals*
  - sandwiches, pasta, cake
16. *seafood*
17. *spices/herbs*
18. *spreads*
19. *vegetables*
20. *ethnic foods*
  - spanish/mexican
    - beans, burrito, quesadilla, rice, and so forth
  - italian
    - pizza, pasta, spaghetti, and so forth
  - other ethnicities not specified
21. *occasions*
  - breakfast foods (time of day)
    - pancakes, waffles, eggs, bacon, cereal, and so forth
  - birthday foods
    - cake, pizza, ice-cream, and so forth.
